# Supplementary material for: A direct RNA-seq-based EBV latency transcriptome offers insights into the biogenesis of EBV gene products
Source: J Gen Virol. 2025 Aug 26;106(8):002134. doi: 10.1099/jgv.0.002134 (PMC12451641; doi:10.1099/jgv.0.002134)
Supplement: Uncited Supplementary Material 1. [file jgv-106-02134-s001.pdf]

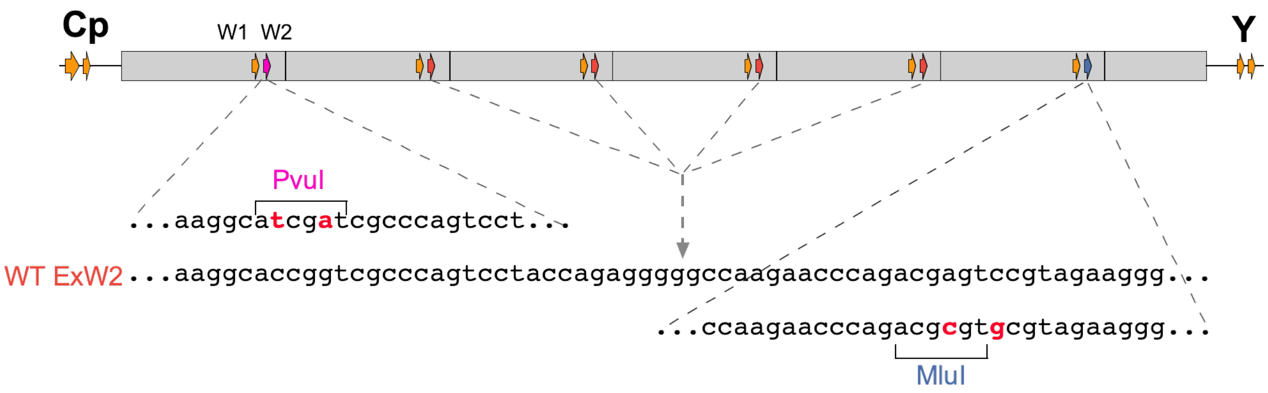

Supporting Figure S1. Barcoding sequences of EBV-BC. Schematic representation of the IR1 repeat indicating the Cp, W1 and Y exons (Orange), with the W2 exons coloured according to their sequence, which is shown below: With the first W2 exon PvuI barcoded (Magenta; top sequence); the next four having Wild-type Exon W2 (Brick Red; middle sequence) and final W2 exon MluI barcoded (Blue; bottom sequence).

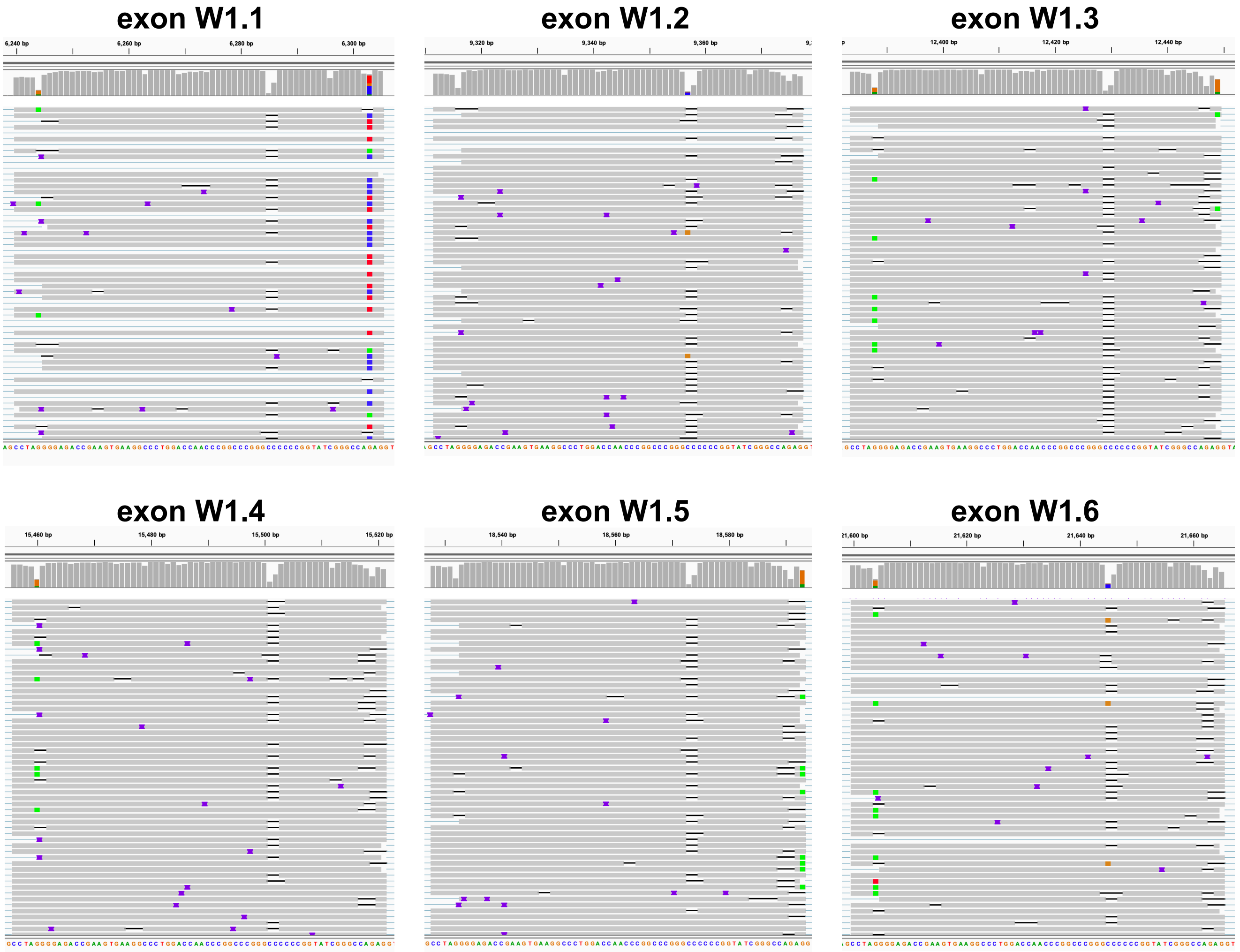

Supporting Figure S2. The STOP codon in the W1 exon of $\mathrm{WT}^{\mathrm{HB9}}$ is in the first exon. Visualisations (using Integrated Genome Browser) of the BAM file of the $\mathrm{WT}^{\mathrm{HB9}}$-LCL reads aligned to the WT^{w} genome, showing each of the six W1 exons, with genome positions (genome position 1 is the Mlu I restriction site in oriP) marked above. Mismatches of reads with consensus sequence are indicated with coloured boxes, and summarised across all reads in the bars at the top. Grey indicates matches to consensus; black line is deletion and blue flanged element insertion, both relative to consensus.

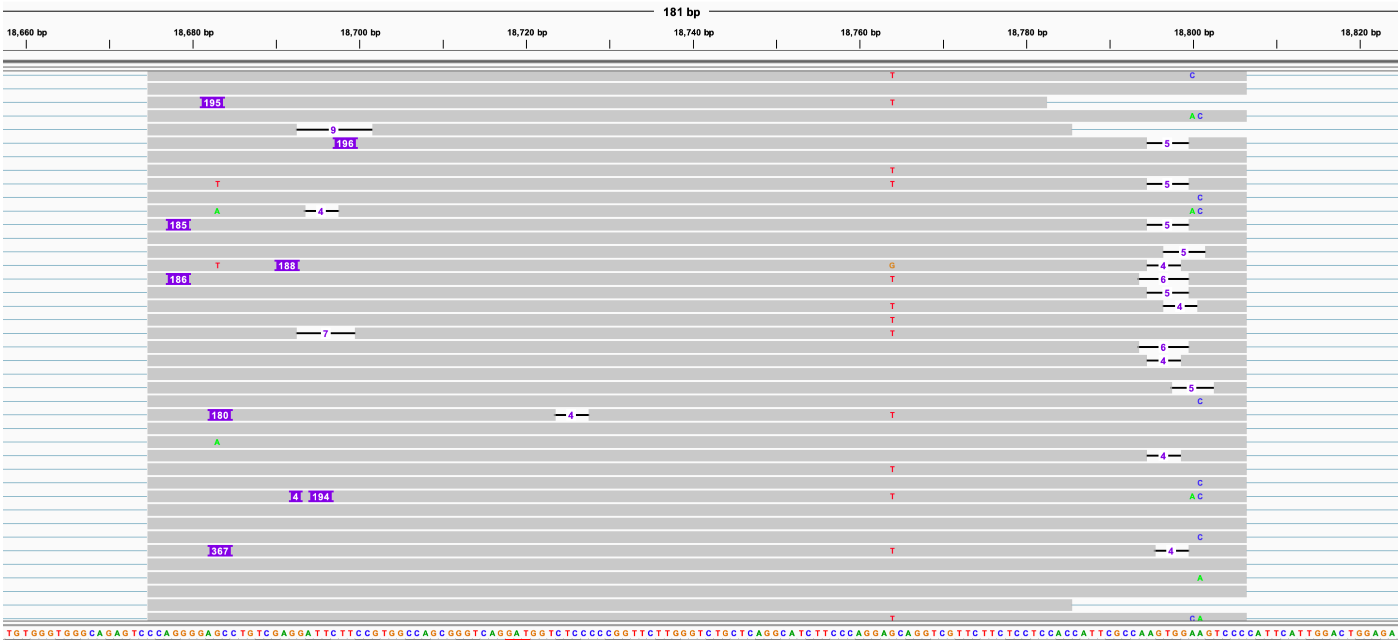

Supporting Figure S3. Misalignment of reads to IR1 is evident as multiple exon pair insertions. Visualisation, using integrated genome browser (indels <4 nt are hidden), of reads aligned to a W2 exon. Un-aligned insertions are indicated by blue bars containing white writing. Expected size of W1-W2 exon pair is 193 nt (W1-W2Δ is 172nt).

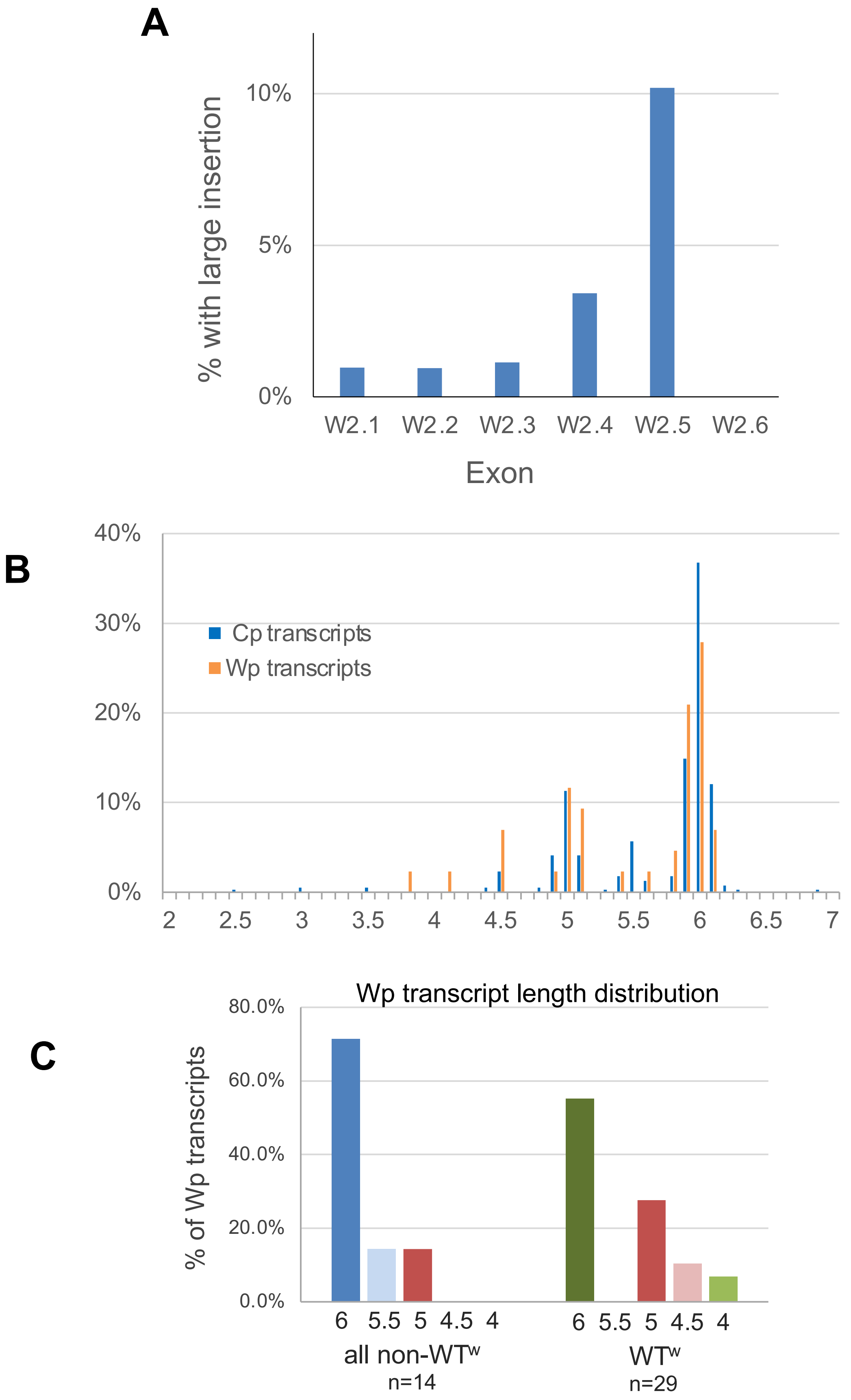

Supporting Figure S4. Validation of IR1 counting strategy. (A) Frequency of mis-mapping in different W2 exons in IR1 (repeat number is after the "W2." Percent with insertion is defined as having a number of nucleotides mapped to the exon that is over 1.5 times the ≈130 nucleotides normally observed aligning to that exon. **(B)** The percentage Cp or Wp transcripts (that also map to at least one canonical exon downstream of IR1) with calculated IR1 repeat numbers, plotted as 0.1 repeat bins (centred on integer numbers). The read numbers cluster around 0.5 repeat values, suggesting this approach is effective at estimating the number of IR1 exons in each transcript. (**C**) The distribution of IR1 length estimates of Wp reads, comparing WT^{w} with the other three samples.
